# Therapeutic assessment of *Nerium oleander*-derived exosome-like nanoparticles against triple-negative breast cancer: a preliminary *in vitro* and network pharmacology approach

**DOI:** 10.3389/fonc.2026.1863223

**Published:** 2026-07-10

**Authors:** Sreyoshi Routh, Tomsy Joseph, Arya Sreekumar, Venkatraman Manickam

**Affiliations:** School of Bio Sciences and Technology, Vellore Institute of Technology, Vellore, Tamil Nadu, India

**Keywords:** apoptosis, cytochrome P450, exosome-like nanoparticles, network pharmacology, TNBC

## Abstract

**Background:**

Triple-negative breast cancer (TNBC) is an aggressive subtype lacking estrogen, progesterone, and HER2 receptors, which restricts targeted therapies and contributes to poor survival. Conventional chemotherapeutics such as doxorubicin and cisplatin show limited efficacy, significant toxicity, and resistance, underscoring the need for alternative strategies. *Nerium oleander*, a traditional medicinal plant, has demonstrated anticancer activity through leaf extracts; however, the role of exosome-like nanoparticles derived from its flowers (NELNs) remains unexplored.

**Methods:**

The therapeutic efficacy of NELNs was evaluated in the MDA-MB-231 TNBC cell line using *in vitro* and *in silico* approaches. Cytotoxicity was assessed by the MTT assay, while AO/EtBr and DCFDA staining examined apoptosis and ROS generation through fluorescence microscopy. Flow cytometry was employed for apoptosis confirmation and cell cycle analysis. Network pharmacology integrating target prediction, PPI network construction, and functional enrichment analysis was performed, followed by molecular docking of NELN-encapsulated metabolites against predicted targets.

**Results:**

NELNs induced significant cytotoxicity, promoted apoptosis, and increased intracellular ROS levels in MDA-MB-231 cells. Flow cytometry confirmed apoptotic induction and revealed alterations in cell cycle progression. Pathway enrichment analysis of previously identified NELN-associated metabolites computationally highlighted arachidonic acid and linoleic acid metabolism, with cytochrome P450 enzymes (CYP1A2, CYP2C9, CYP3A4) emerging as putative targets. Molecular docking predicted favorable binding interactions of NELN metabolites to these targets.

**Conclusion:**

This study provides preliminary evidence that flower-derived NELNs exerted antiproliferative and pro-apoptotic activity against MDA-MB-231 cells under the tested experimental conditions. Furthermore, network pharmacology analyses generated putative mechanistic insights into pathways relevant to TNBC.

## 1 Introduction

Breast cancer is one of the most prevalent cancers among women, particularly in nations with higher human development indices (1). According to GLOBOCAN 2022 estimates, by 2050, there would be approximately 1.4 million new cases of breast cancer and 0.5 million fatalities in Asia, with China and India accounting for most of the new cases and deaths (1). Globally, breast cancer poses a significant health challenge due to its higher incidence, mortality, and considerable biological diversity. Clinically, breast cancer is divided into several biological subtypes, including luminal A, luminal B, HER2-enriched, and basal-like, each with distinct prognostic and therapeutic characteristics. Triple-negative breast cancer (TNBC) constitutes approximately 20% of all breast cancers (2). It is a basal-like, heterogeneous, and aggressive type characterized by the absence of estrogen receptor (ER), progesterone receptor (PR), and human epidermal growth factor receptor 2 (HER2) expression. Moreover, it is more common in younger and premenopausal women, is physiologically aggressive, and is often linked to a worse prognosis (3). Primarily, BRCA1 gene mutation represents one of the primary genetic alterations in TNBC, with germline mutations in BRCA1 or BRCA2 (crucial for homologous recombination DNA repair) detected in approximately 10%–20% of cases (3). Other frequently mutated genes in TNBC include TP53 (present in 60%–70% of TNBCs) and PIK3CA and genes associated with cell cycle regulation and DNA damage response such as CCNB1, CCNA2, CDK1, and CDK2 (4–7). Accumulation of such genetic mutations leads to genomic instability and defective DNA repair mechanisms, promoting carcinogenesis (8–10). Unlike other cancer types, TNBC lacks effective hormone therapy or medications targeting receptor proteins (like HER2) and other targeted therapies, leaving chemotherapy as the primary form of treatment (11). Most used chemotherapeutic agents mainly include anthracyclines (doxorubicin and epirubicin), taxanes (paclitaxel and docetaxel), and metal-based compounds (carboplatin and cisplatin), often administered in combination regimens to maximize efficacy in TNBC treatment (12–15). Unfortunately, due to limited availability of FDA-approved medications and lack of combination therapies, patients frequently suffer from severe side effects associated with chemotherapy; also, the conventional treatment strategies often fail to yield satisfactory clinical outcomes, as certain tumors are either inherently resistant or rapidly acquire resistance to available drugs (16). Also, anthracycline and taxane-based chemotherapy for TNBC have been reported to cause myelosuppression, irreversible cardiotoxicity, alopecia, nausea, and vomiting, which adversely affect patients’ quality of life and can limit treatment adherence (12, 15). Due to the existing obstacles and constraints of traditional anticancer therapies, there is a growing interest in nature-based therapies because of their reduced side effects, rendering them a better and feasible option in cancer treatment (17).

Interestingly, exosome-like nanoparticles (ELNs) derived from plants and plant products (e.g., flowers, fruits, and vegetables) have garnered significant attention in the era of nature-based therapies owing to their enhanced biocompatibility and lower immunogenicity. For instance, *Acorus calamus*-derived exosome-like nanoparticles (ACELNs) have been specifically investigated for their cytotoxic and apoptosis-inducing effects in breast cancer models, including the TNBC cell line MDA-MB-231 (18, 19). Plant-derived ELNs can encapsulate diverse cargoes, including DNA, RNA, proteins, lipids, and metabolites, exerting functional roles when transported to recipient cells. For instance, ELNs derived from *Brassica javanica* have demonstrated efficacy in breast cancer treatment, primarily through the activity of their microRNA content (20). Furthermore, ELNs derived from other edible plants, including *Citrus limon* L.-derived extracellular nanovesicles (CLENs) (21), tea flower-derived nanovesicles (TLNT) (22), nanovesicles derived from the seeds of *Elettaria cardamomum* (23), and several other plant sources, have also been recognized as emerging natural nanotherapeutics with significant promising results for TNBC treatment. Additionally, plant-derived ELNs combined with chemotherapeutic agents in dual-delivery systems have exhibited enhanced efficacy in preclinical models by improving drug delivery and exerting synergistic antitumor effects (21).

Intriguingly, plants from the Apocynaceae family have demonstrated considerable potential in TNBC treatment attributed to their diverse bioactive compounds, especially alkaloids, flavonoids, and terpenoids with potent anticancer properties (24, 25). Flowers such as *Catharanthus roseus* belonging to the Apocynaceae family secretes vinca alkaloids such as vinblastine and vincristine, which are clinically used as chemotherapeutic agents against breast cancer, including TNBC, by inducing cell cycle arrest and apoptosis (26, 27). Similarly, *Nerium oleander* L. from the Apocynaceae family is a shrub growing in subtropical regions, which is used as a medicinal herb in Indian Ayurveda and Unani (27). In the Near East and Southern Asia, it has also been used in traditional medicine as anti-inflammatory, antidiabetic, and anticancer herbal medicine, as well as against indigestion, malaria, leprosy, and mental diseases (28–30). *Nerium oleander* leaf extracts have shown strong antiproliferative effects against TNBC cell lines owing to the presence of cardenolides and triterpenoids, which impede cancer cell development and migration (24). Nevertheless, the therapeutic implications of *N. oleander* L. flowers and their derived exosome-like nanoparticles (NELNs) in breast cancer have not yet been adequately investigated. Therefore, the present study is aimed to assess the biological activity of NELNs against triple-negative breast cancer cells and examine their potential therapeutic application through comprehensive *in vitro* and *in silico* analyses. Therefore, the present study aimed to evaluate the anticancer potential of NELNs and investigate their potential as a novel biotherapeutic for TNBC through comprehensive *in vitro* and *in silico* analyses. We have assessed the therapeutic significance of NELNs *in vitro* utilizing the commonly used TNBC cell line MDA-MB-231. The antiproliferative effects of NELNs are systematically evaluated to determine their functional influence on TNBC cells. Furthermore, to delineate the preliminary mechanistic insights into the molecular activity potentially associated with NELNs in TNBC, metabolites previously identified from NELN through GC–MS analysis were used for network pharmacology and molecular docking analyses.

## 2 Materials and methods

### 2.1 Collection of flowers, isolation, and characterization of NELNs

Fresh flowers were collected from Katpadi, Tamil Nadu, in the month of August 2024 and were authenticated as *N. oleander* by Dr. D. Stephen, Assistant Professor, American College, Madurai, Tamil Nadu. The fresh petals were separated, washed with distilled water, and blended with 1× phosphate buffered saline (PBS) in a 1:3 ratio using an electric grinder until a homogeneous mixture was attained. The homogeneous mixture was later passed through a Whatman filter paper, and the filtrate was subjected to a series of centrifugation steps to isolate the NELNs. Initially, the filtrate was centrifuged at 1,000*g* for 10 min (4 °C) followed by 6,000*g* for 20 min (4 °C) to remove the big debris. Subsequently, the supernatant was collected and centrifuged at 10,000*g* for 30 min (4 °C) to remove the pellet containing smaller cell debris. Furthermore, the supernatant was collected, filtered through a syringe filter (pore size: 0.22 μm) twice, and subjected to ultracentrifugation (model name: Optima MAX-XP, Beckman Coulter, Brea, CA, USA) (rotor type: TLA-110 fixed angle rotor, Beckman Coulter, Brea, CA, USA) at 150,000*g* for 90 min (4 °C). The pellet was collected, washed with 1× PBS to remove any existing contaminants, and further subjected to ultracentrifugation (150,000*g* for 90 min at 4 °C) (31). The supernatant was removed, and the pellet was suspended in syringe-filtered 1× PBS (pore size: 0.22 μm) and further subjected to physical characterization. For determining the particle size and distribution, NELN suspensions were prepared in appropriate dilutions with syringe-filtered 1× PBS and characterized using nanoparticle tracking assay (NTA) through a Particle Matrix ZetaView PMX 130-Mono laser. Furthermore, to analyze the stability of NELNs in suspension, zeta potential was measured using Particle Matrix ZetaView PMX 130-Mono laser, and physical morphology was assessed using a field emission scanning electron microscope (FE-SEM). For the visualization of NELN morphology through FE-SEM, briefly, the NELNs were diluted 10-fold using syringe-filtered 1× PBS. A total of 100 μL of the diluted sample was placed onto a cover slip and fixed in 2.5% glutaraldehyde. Subsequently, the sample was dehydrated using a gradient of ethanol washes (30%, 50%, 80%, 90%, and 100%) and visualized under FE-SEM (model name: FEI QUANTA 250 FEG, Thermo Fishe, Bangalore, India) (32).

### 2.2 Cell viability assay of NELNs

The MTT (3-(4,5-dimethylthiazol-2-yl)-2,5-diphenyltetrazolium bromide) assay was performed to assess the anticancer activity of NELNs *in vitro*. The triple-negative breast cancer cell line MDA-MB-231 and the non-cancerous cell line HEK239T (human embryonic kidney cell line) were cultured in Dulbecco’s modified Eagle medium (DMEM), with 10% fetal bovine serum (FBS) and 1% antibiotic–antimycotic solution and maintained in a humidified condition at 5% CO_2_, 37 °C. Upon reaching 80% confluency, the cells were trypsinized, seeded in a 96-well plate (1 × 10^4^ cells), and incubated at 37°C, 5% CO_2_ for 24 h. NELN concentrations used in all the *in vitro* experiments are expressed as µg/mL of total vesicular protein. Protein concentration was determined using a bicinchoninic acid (BCA) assay according to the manufacturer’s instructions, and treatment concentrations were prepared based on the measured protein content (33). The seeded cells were further treated with different concentrations of NELNs and incubated under the same conditions for the next 24 h (34). Post-treatment, the media was removed and MTT (5 mg/mL) was added to the treated cells and incubated for 4 h to allow the formation of formazan crystals. Subsequently, the MTT solution was removed, DMSO (100 μL) was added, and OD (optical density) was measured using a 96-well plate reader (model: Agilent BioTek Epoch). Furthermore, the IC_50_ for NELNs was determined from cell viability data using non-linear regression analysis. Cell viability values were normalized to the untreated control group. A dose–response curve was generated and the IC_50_ value was calculated from the fitted curve.

### 2.3 Assessment of intracellular ROS generation

To estimate the percentage of intracellular ROS generation in MDA-MB-231 cells, DCFDA (2′,7′-dichlorodihydrofluorescein diacetate) was used. Briefly, MDA-MB-231 cell lines were maintained in DMEM with 10% FBS and 1% antibiotic–antimycotic solution in a humidified condition at 37°C, 5% CO_2_, and upon reaching 80% confluency, the cells were further seeded in a 6-well plate (1 × 10^5^ cells) and incubated at 37 °C, 5% CO_2_ for 24 h. The cells were further treated with NELNs and positive control doxorubicin (2 μg/mL) incubated for the next 24 h. To quantitatively estimate the percentage of intracellular ROS generation through flow cytometry, cells were washed twice with 1× PBS and trypsinized post-treatment. Following trypsinization, the cells were further collected, washed with 1× PBS, and incubated with 20 μM of DCFDA for 20 min in the dark (35). Post-incubation, the percentage of intracellular ROS generation was determined using a CytoFlex S flow cytometry (model name: Beckman Coulter, Brea, CA, USA).

### 2.4 Determination of apoptosis

Apoptosis was evaluated in MDA-MB-231 cells using Annexin-V FITC/propidium iodide (PI) staining using flow cytometry and acridine orange/ethidium bromide (AO/EtBr) dual staining using an EVOS fluorescence microscope. Briefly, MDA-MB-231 cells were seeded into a 6-well plate (1 × 10^5^ cells) and incubated overnight. Furthermore, the cells were treated with NELNs and positive control doxorubicin (2 μg/mL) and incubated for the next 24 h. The percentage of apoptotic cells was detected using Annexin V-FITC/PI apoptosis kit (Elabscience, Wuhan, China) following the manufacturer’s protocol. Post-treatment, the cells were washed twice using 1× PBS, trypsinized, collected, and resuspended in 500 μL of 1× Annexin V binding buffer. Furthermore, 5 μL of Annexin V-FITC and PI were added to the resuspended cells, vortexed for 15 s, and incubated in the dark for 15–20 min. The cells were analyzed immediately using a CytoFlex S flow cytometry (model name: Beckman Coulter, Brea, CA, USA) at 518 nm for Annexin V and 620 nm for PI (36). For the analysis, cells were initially gated using forward scatter (FSC) and side scatter (SSC) parameters to exclude cell debris. Doublet discrimination was subsequently performed using FSC-A versus FSC-H gating to ensure analysis of single-cell populations. Furthermore, fluorescence compensation was applied to minimize spectral overlap between FITC and PI channels. Cell populations were further classified into four quadrants: viable cells (Annexin V−/PI−), early apoptotic cells (Annexin V+/PI−), late apoptotic cells (Annexin V+/PI+), and necrotic cells (Annexin V−/PI+).

Apoptotic cells were further visualized using the AO/EtBr dual staining method through the fluorescence microscope. MDA-MB-231 cells were seeded into a 6-well plate, treated with NELNs, and incubated overnight. For microscopic visualization of apoptotic and necrotic cells, the cells were washed with 1× PBS twice after treatment. Furthermore, cells were stained with 50 μL of AO/EtBr (1:1) and visualized using the fluorescence microscope (model: EVOS M5000, Invitrogen, Bangalore, India) (37).

### 2.5 Analysis of cell cycle distribution

Cell cycle distribution in MDA-MB-231 was analyzed using flow cytometry. Briefly, the cells were seeded into a 6-well plate and maintained overnight at 37°C, 5% CO_2_. Cells were subsequently treated with NELNs and positive control doxorubicin (2 μg/mL) and incubated for the next 24 h. Post-treatment, the cells were washed twice with 1× PBS, harvested through trypsinization, fixed in 70% ethanol, and stored at −20°C for 24 h. The fixed cells were then stained with 50 μg/mL of PI and incubated in the dark for 20 min (38, 39). Furthermore, the cells were analyzed using CytoFlex S flow cytometry (Model name: Beckman Coulter, Brea, CA, USA), and the percentage of cells accumulated at different phases of the cell cycle was recorded.

### 2.6 Statistical analysis

All the experiments were performed using three independent biological replicates, and data were presented as mean ± standard deviation (SD). Statistical analysis was performed using GraphPad Prism 8.0. To analyze the comparisons among multiple groups, one-way ANOVA with Tukey’s multiple comparison test was utilized and *p <*0.05 was considered statistically significant.

### 2.7 Network pharmacology

#### 2.7.1 Target prediction of NELN-encapsulated metabolites

The phytochemical composition of the NELN-encapsulated metabolites was previously characterized using GC–MS analysis in our study (40), and the retrieved 21 metabolites were included in the computational workflow and used as a compound library for target prediction. Briefly, isomeric SMILES of the NELN metabolites were retrieved from the PubChem database and used for prediction of their targets using SwissTargetPrediction (STP) and the similarity ensemble approach (SEA) with *Homo sapiens* as the target species, and the targets predicted with non-zero prediction probability were retained (41, 42). Targets for all the metabolites were retrieved, duplicate entries were removed, and a Venn diagram was generated to illustrate the overlapping SEA–STP targets using the VENNY 2.1.0 webtool.

#### 2.7.2 Collection of TNBC relevant targets

Targets relevant to TNBC were collected from multiple databases such as DisGeNET, GeneCards, and NCBI enabling the *H. sapiens* filter (43, 44). The retrieved targets were combined, duplicate entries were removed and further cross-referenced with the overlapping SEA–STP targets of NELN metabolites, and a Venn diagram was constructed using the VENNY 2.1.0 webtool to obtain the common targets of the NELN-encapsulated metabolites relevant to TNBC.

#### 2.7.3 Construction of the protein–protein interaction network

To visualize the interaction among all the targets, a protein–protein interaction (PPI) network was constructed using STRING 12.0 (online database) with “*H. sapiens*” as the selected organism and a medium confidence threshold score of >0.500 (45). The interactions were derived from integrated STRING sources, including experimental data and curated databases. The nodes, which were disconnected (without any interaction edges) from the network, were removed, and the final output file was exported to Cytoscape 3.10.0 to visualize the final interaction network (46, 47). Furthermore, hub targets were identified based on network topological analysis using the CytoHubba plugin in Cytoscape, and the top 15 hub targets were selected based on closeness parameter.

#### 2.6.4 Gene ontology and enrichment analysis

To uncover the biological significance of the hub targets in TNBC, gene ontology (GO) and enrichment analysis were performed. The identified hub targets were subjected to GO analysis through the ShinyGO webtool (https://bioinformatics.sdstate.edu/go) using *H. sapiens* as the background species (48, 49). It was further classified into three categories, namely, molecular functions (MF), biological processes (BP), and cellular component (CC), and the GO terms with *p*-value <0.05 and false discovery rate (FDR) <0.05 were considered significantly enriched. To further investigate the biological pathways associated with the hub targets in modulating TNBC, pathway enrichment analysis was performed using the Kyoto Encyclopedia of Genes and Genomes (KEGG) database through the ShinyGO webtool. Pathways with adjusted *p*-value <0.05 were considered significantly enriched.

#### 2.6.5 Molecular docking

Three target proteins, CYP1A2 (PDB ID: 2HI4), CYP3A4 (PDB ID: 1TQN), and CYP2C9 (PDB ID: 4NZ2), were selected for docking. The 3D crystal structures of the proteins were downloaded from the RCSB PDB database (https://www.rcsb.org/). The PyMol software application (version 3.1.5.1) (50) was used to add hydrogens and remove the existing water molecules and ligands, and it was saved in pdbqt format. Discovery Studio (51) was used to predict the active site coordinates of the targets: CYP1A2 (6.1836, 25.8174, 21.1228), CYP2C9 (−52.8729, −44.0568, −22.201), and CYP3A4 (−13.5018, −19.6332, −10.6856). Molecular docking was performed for the 21 bioactive compounds encapsulated in NELNs with the three target proteins to verify their binding affinities using AutoDock Vina in Swiss dock platform (52, 53). The targets in pdbqt were imported to the platform, and the isomeric SMILES of the compounds were entered. The active site coordinates of the targets were defined according to the proteins. The resulting docking scores or binding energy after docking was used to represent the docking results (54).

## 3 Results

### 3.1 Characterization of isolated NELNs

**Figure 1:**
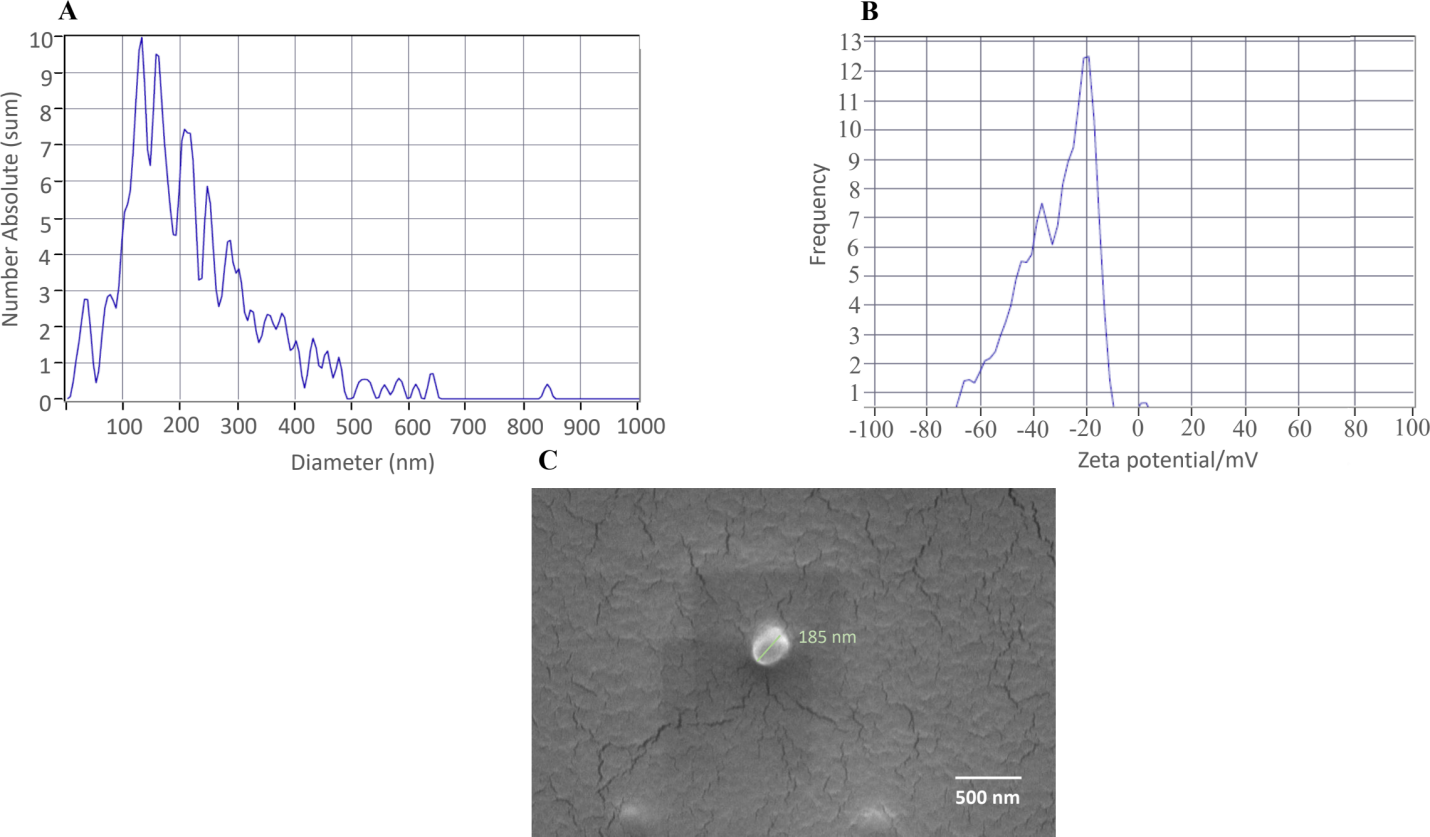
Determination of **(A)** size distribution and particle concentration of NELNs using NTA. **(B)** Zeta potential of NELNs. **(C)** Morphological visualization of NELNs using FE-SEM (scale bar: 500 nm). Data are representative of three independent experiments.

NELNs were freshly isolated using the ultracentrifugation method, and their physicochemical properties were characterized to confirm their size, structure, and stability. NTA analysis revealed the size distribution of NELNs predominantly ranging between 50 and 200 nm (diameter) with particle concentration of 5.3 × 10^13^ particles/mL depicted in **Figure 1A**. To analyze the colloidal stability of NELNs in solution, surface charge was measured. Zeta potential analysis revealed the surface charge of NELNs ranging between −20 and −40 mV (**Figure 1B**). Furthermore, morphological assessment of NELNs through FE-SEM confirmed a spherical vesicular structure of NELNs with a particle diameter of 185 nm (**Figure 1C**).

### 3.2 Assessment of cytotoxicity of NELNs

**Figure 2:**
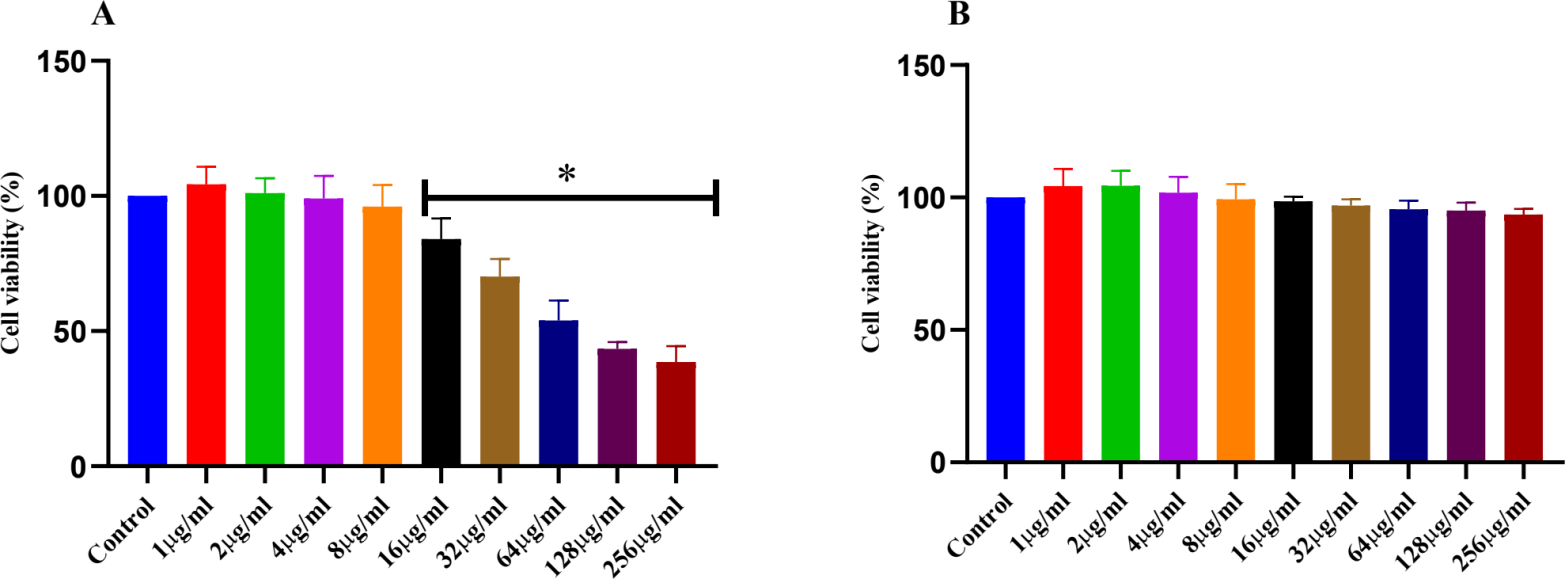
Cytotoxicity activity of NELNs. **(A)** MDA-MB-231 and **(B)** HEK293T cells. Cells were treated at different concentrations (1, 2, 4, 8, 16, 32, 64, 128, and 256 μg/mL) for 24 h, and cell viability was assessed using the MTT assay. Data are presented as *n* = 6, mean ± SD, *p < 0.05.

NELN cytotoxic activity was evaluated in the TNBC cell line MDA-MB-231 and the non-tumorigenic cell line HEK293T using the MTT assay. The cells were treated at varied concentrations of NELNs for 24 h and assessed for cytotoxic activity. NELN treatment in the MDA-MB-231 cell lines exhibited a dose-dependent cytotoxicity with minimal toxicity at lower concentrations and a significant reduction in cell viability at concentrations ranging between 16 and 256 μg/mL (**Figure 2A**). The IC_50_ for NELNs was determined from cell viability data using non-linear regression analysis. Cell viability values were normalized to the untreated control group. A dose–response curve was generated, and the IC_50_ value was calculated at 64 μg/mL from the fitted curve. Furthermore, biocompatibility of NELNs was assessed in the HEK293T cell line using the MTT assay, and the cells did not exhibit any significant cytotoxicity at various concentrations of NELNs as depicted in **Figure 2B**.

### 3.3 Determination of intracellular ROS generation in MDA-MB-231 cells

**Figure 3:**
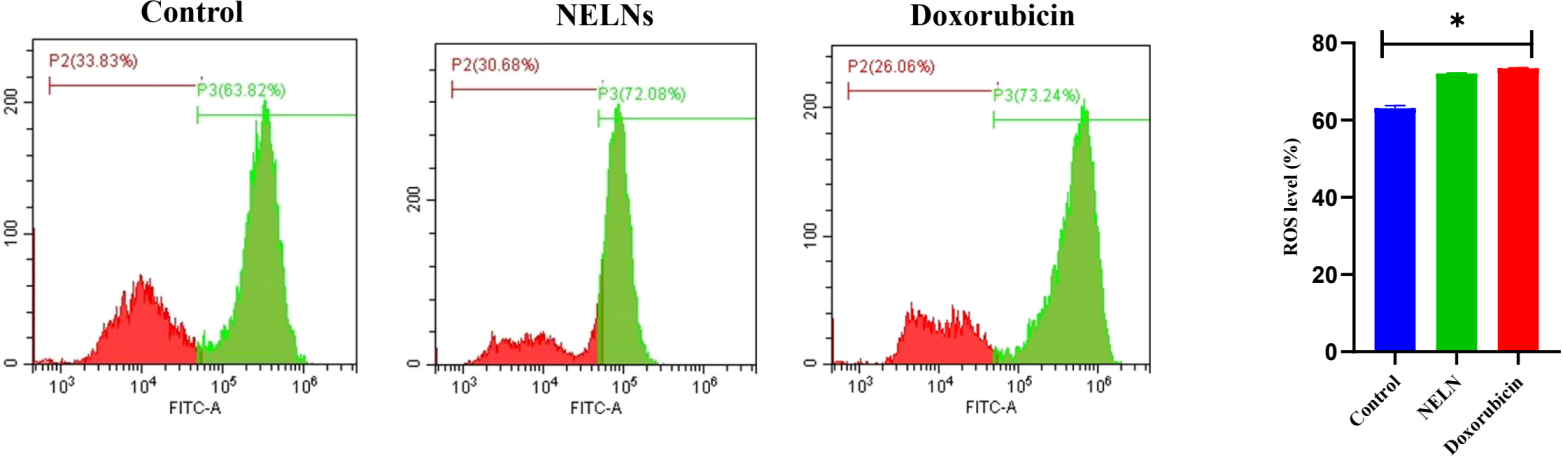
Determination of intracellular ROS generation using DCFDA staining in untreated, NELN-treated (64 μg/mL), and doxorubicin-treated (2 μg/mL) MDA-MB-231 cells for 24 h. Data are presented as *n* = 3, mean ± SD, *p < 0.05.

The effect of NELNs on intracellular ROS generation was measured in MDA-MB-231 cells using DCFDA staining through flow cytometry. As depicted in **Figure 3**, the NELNs and doxorubicin (positive control) exhibited a significant increase in intracellular ROS levels (in percentage) compared to the control cells. Doxorubicin was used as a reference drug in the present study.

**Figure 4:**
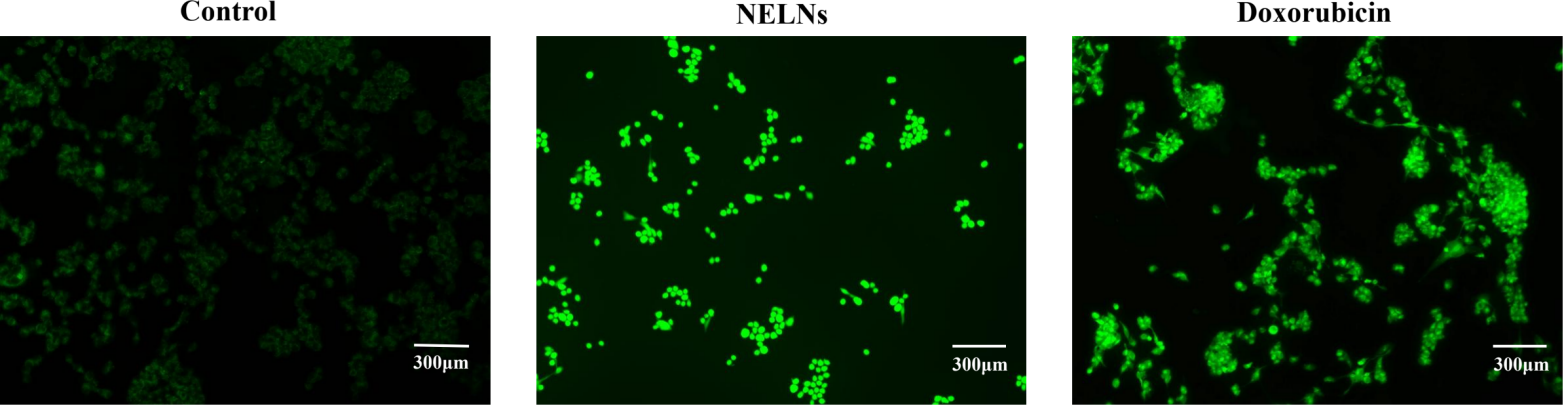
Visualization of intracellular ROS generation using DCFDA staining in untreated, NELN-treated (64 μg/mL), and doxorubicin-treated (2 μg/mL) MDA-MB-231 cells for 24 h. Data are indicative of *n* = 3.

Furthermore, the intracellular ROS generation was qualitatively assessed in MDA-MB-231 cells using DCFDA staining using the fluorescence microscope. As represented in **Figure 4**, control cells resulted in weaker green fluorescence, indicating a basal level of ROS generation. However, there was a marked increase of fluorescence in NELN- and doxorubicin-treated cells, depicting enhanced ROS production compared to the control group.

### 3.4 Evaluation of apoptosis in MDA-MB-231 cells

**Figure 5:**
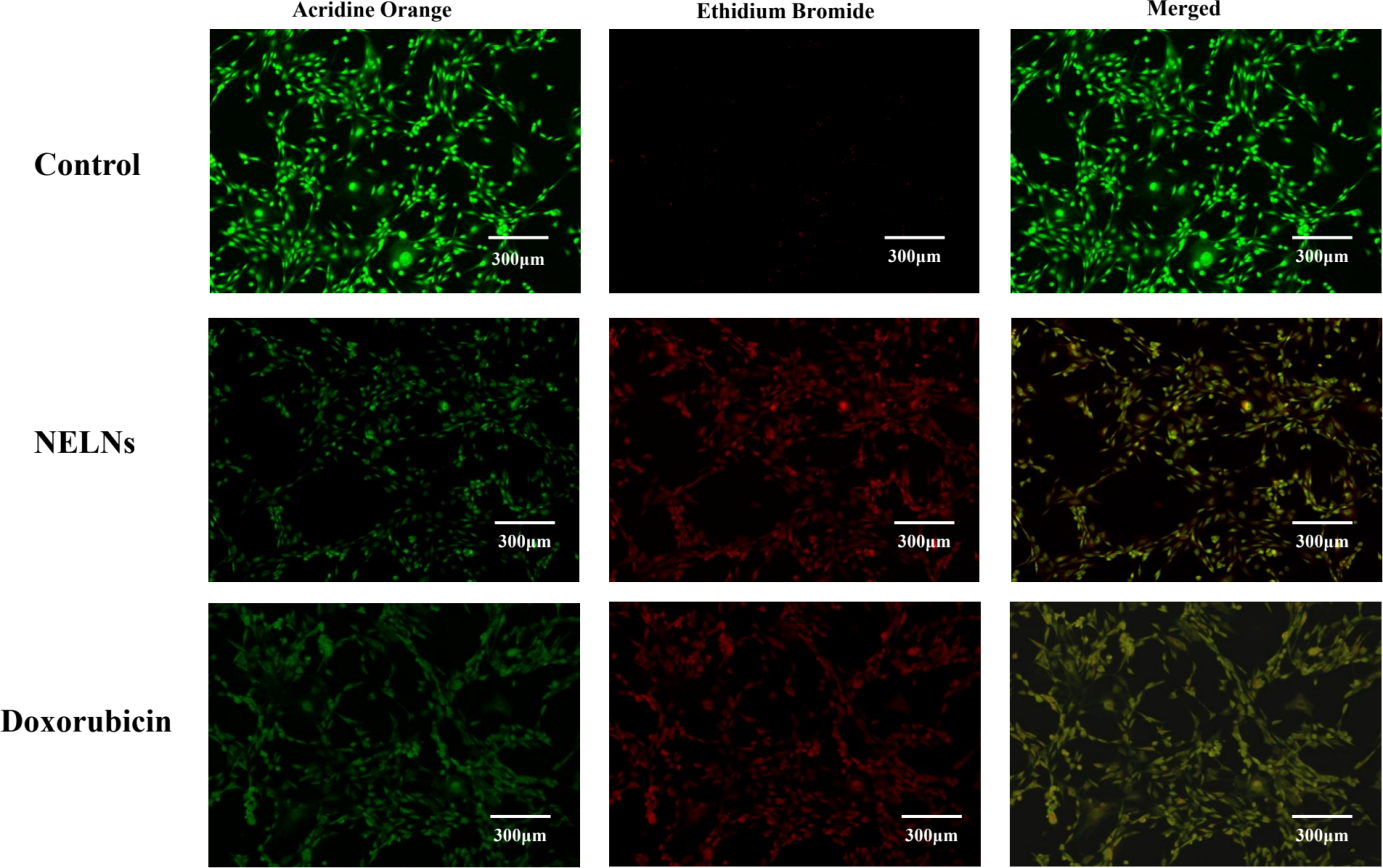
Determination of apoptosis using AO/EtBr dual staining through fluorescence microscope in the control, NELN-treated (64 μg/mL), and doxorubicin-treated (2 μg/mL) cells (24 h). Data are representative of three independent experiments.

The effect of NELNs on cellular apoptosis and necrosis was assessed in MDA-MB-231 cells using AO/EtBr dual staining through the fluorescence microscope. As depicted in **Figure 5**, control cells predominantly exhibited green fluorescence indicative of viable cells with the cell membrane intact. However, in the NELN- and doxorubicin-treated groups, we observed an increased number of apoptotic cells characterized by yellow to orange fluorescence as observed in the merged section compared to the control cells.

**Figure 6:**
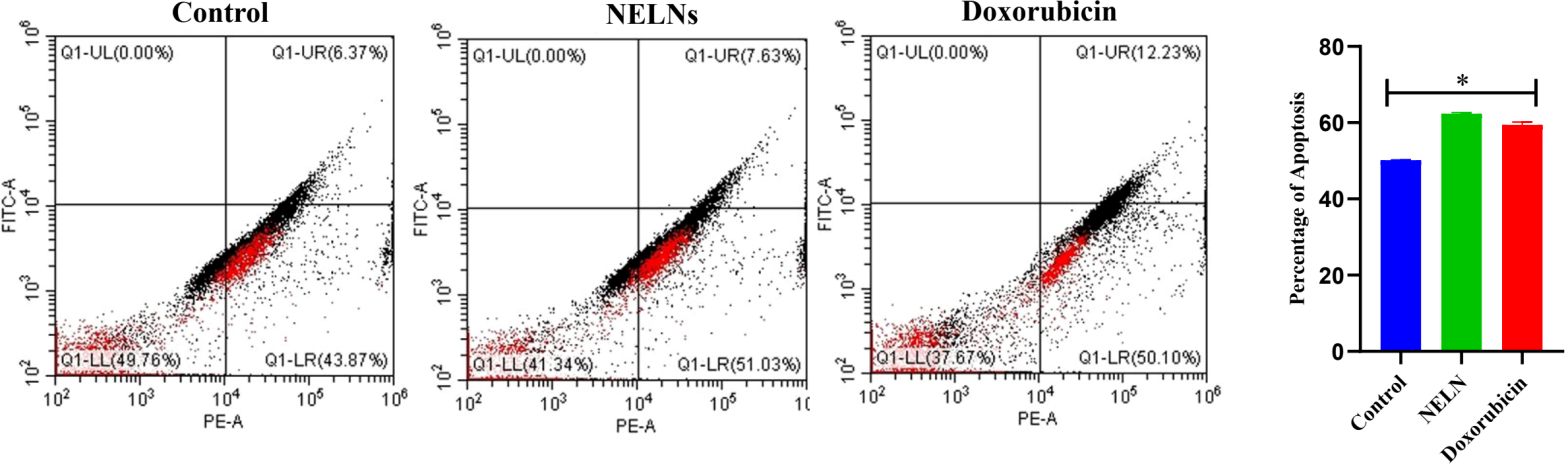
Assessment of apoptosis using Annexin-PI through flow cytometry in the control, NELN-treated (64 μg/mL), and doxorubicin-treated (2 μg/mL) MDA-MB-231 cells for 24 h. Data are presented as *n* = 3, mean ± SD, *p < 0.05.

Furthermore, quantitative estimation of apoptosis was performed using Annexin V-FITC/PI staining through flow cytometry (**Figure 6**). Representative quadrant distributions corresponding to viable cells (Q1-LL), early apoptotic cells (Q1-LR), late apoptotic cells (Q1-UR), and necrotic cells (Q1-UL) were analyzed in the control, NELN-, and doxorubicin-treated groups. In the untreated control group, viable cells accounted for 49.76% of the total population, while the early apoptotic and late apoptotic populations represented 43.87% and 6.37%, respectively, with negligible necrotic cells. The comparatively elevated basal apoptotic fraction observed in untreated cells may partially reflect the intrinsic biology of MDA-MB-231 cells, which are characterized by elevated oxidative stress, high metabolic activity, and genomic instability under *in vitro* culture conditions. Upon NELN treatment, the viable cell population decreased to 41.34%, whereas early apoptotic and late apoptotic populations increased to 51.03% and 7.63%, respectively, resulting in a total apoptotic population of 58.66%. Similarly, doxorubicin treatment reduced viable cells to 37.67% and increased early and late apoptotic populations to 50.10% and 12.23%, respectively, corresponding to 62.33% total apoptotic cells. Necrotic cell populations remained negligible across all experimental groups. Collectively, both NELN and doxorubicin treatments demonstrated increased Annexin V-positive cell populations relative to untreated controls, indicating enhanced apoptotic response in MDA-MB-231 cells under identical experimental conditions.

### 3.5 Analysis of cell cycle distribution

**Figure 7:**
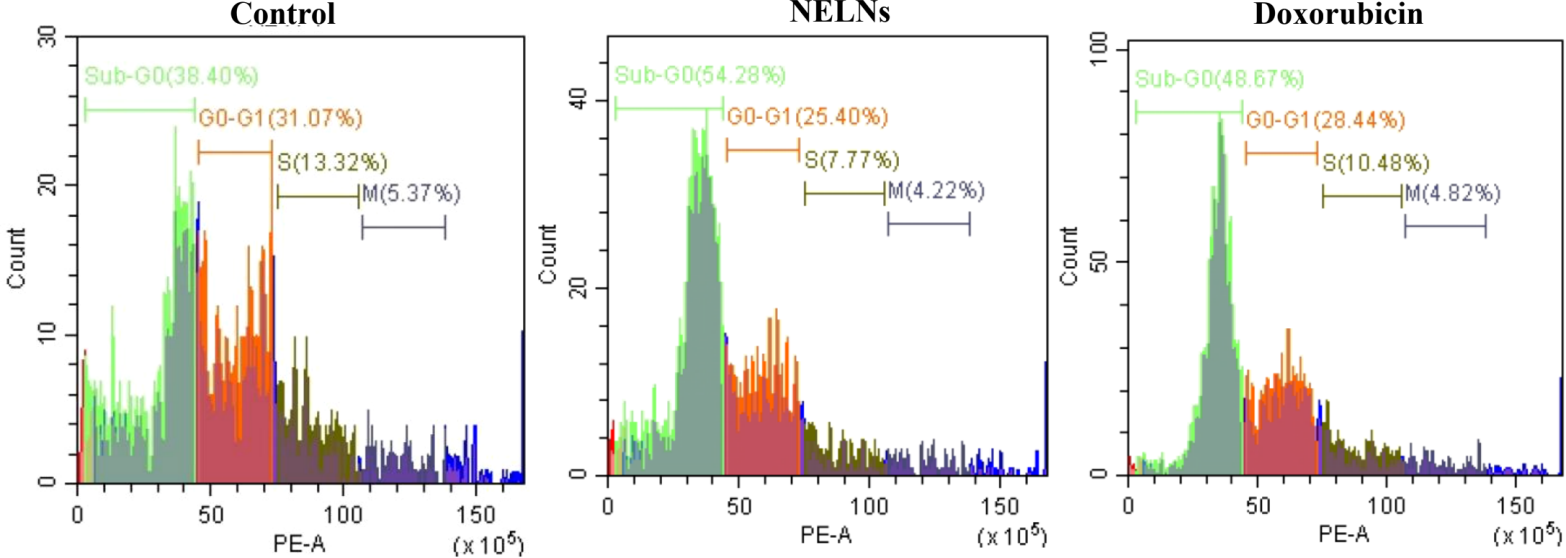
Analysis of cell cycle arrest through flow cytometry in the control, NELN-treated (64 μg/mL), and doxorubicin-treated (2 μg/mL) MDA-MB-231 cells (24 h). Data are representative of three independent experiments.

The effect of NELN treatment on cell cycle distribution was assessed in MDA-MB-231 cells by quantifying the DNA content in each cell cycle stage through flow cytometry (**Figure 7**). Cell populations present across different cell cycle stages such as sub-G0, G0–G1, S, and M were estimated in untreated and treated conditions. As evidenced in **Figure 7**, cells in the control group predominantly occupied the G0–G1 stage (31.07%), followed by the S (13.32%) and M (5.37%) phases. Notably, upon NELN and doxorubicin treatment, we observed a reduction in cell accumulation in G0–G1, S, and M phases compared to control, thus demonstrating the potential of NELNs in modulating the cell cycle distribution in MDA-MB-231 cells.

### 3.6 Target prediction of NELN metabolites relevant to TNBC

**Figure 8:**
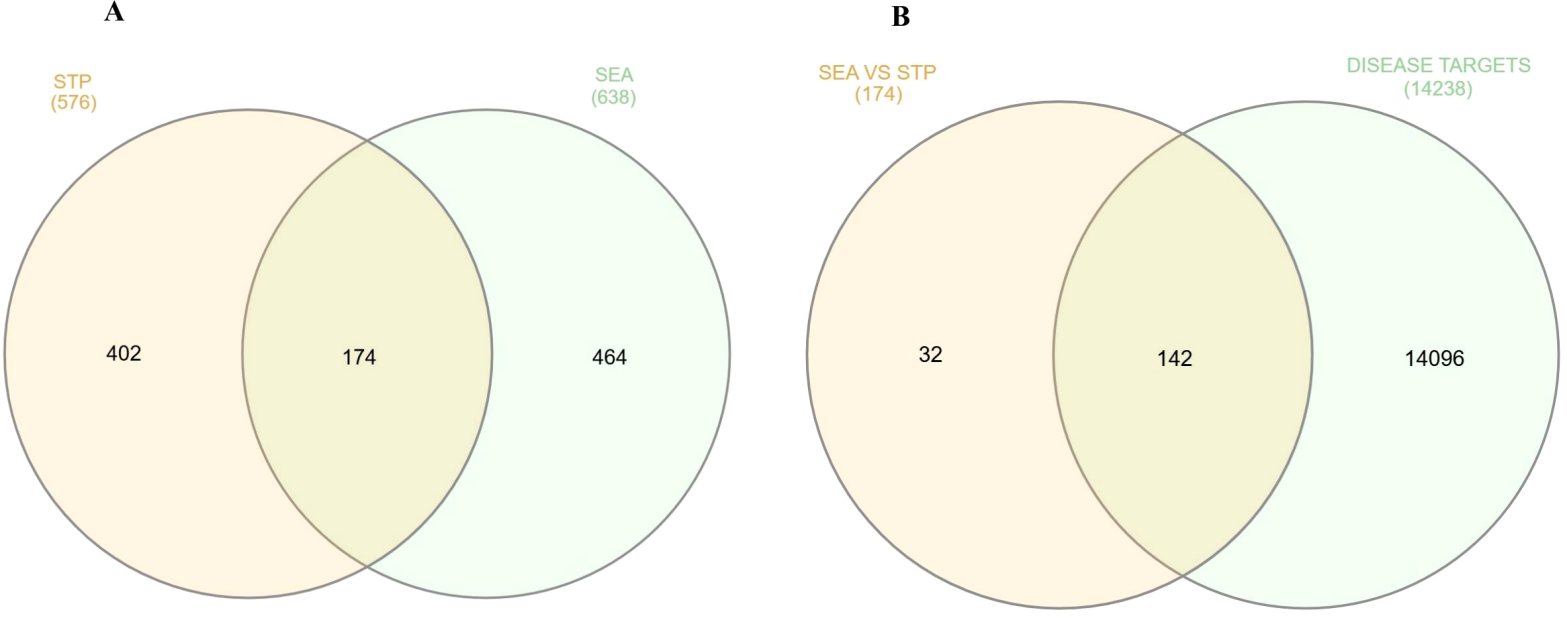
Target prediction using the VENNY 2.1.0 webtool. **(A)** STP–SEA overlapping targets. **(B)** SEA–STP and TNBC overlapping targets.

To further delineate the molecular targets potentially underlying the biological activity of NELN in TNBC treatment, a systematic target prediction and overlapping analysis was performed using the VENNY webtool. Metabolites previously detected in NELNs through GC–MS analysis in our previous work (40) were curated and used as a computational input library for target prediction and network pharmacology analyses. The chemical structures for each of the metabolites were retrieved from the PubChem database, and the canonical SMILES were used for target prediction of the metabolites. Initially, 576 and 638 putative targets for NELN metabolites were retrieved from STP and SEA, respectively, and overlapping analysis revealed 174 SEA–STP common targets (**Figure 8A**). Furthermore, to analyze the disease relevance of the NELN metabolites, TNBC-relevant targets (14,238) were retrieved from three different databases (**Figure 8B**).

### 3.7 PPI network analysis and hub gene identification

**Figure 9:**
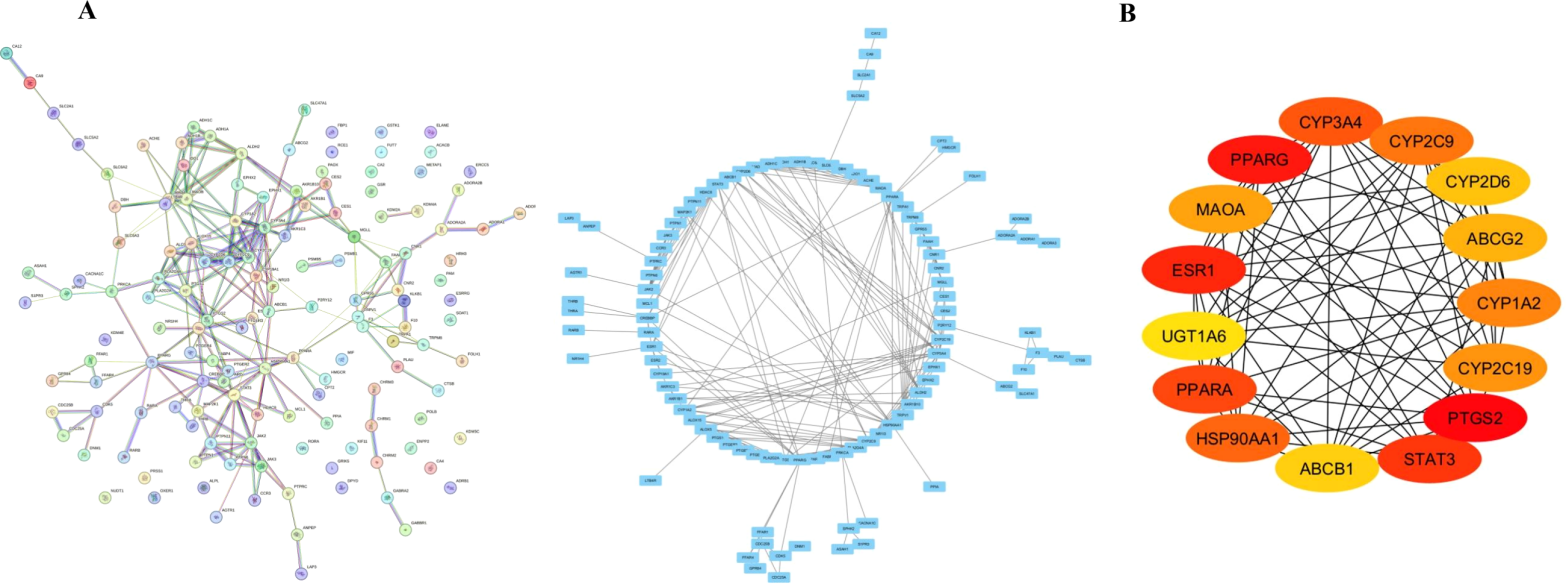
PPI network and hub gene identification. **(A)** PPI network construction of 142 SEA–STP and TNBC overlapping targets exhibiting 142 nodes and 750 edges with an average local clustering coefficient of 0.452 using the STRING database and Cytoscape. **(B)** Top 15 hub target identification using Cytoscape.

To elucidate the molecular mechanisms underlying the potential of NELNs in TNBC treatment, a PPI network was constructed for the intersected 142 targets of NELNs and TNBC using the STRING database and visualized using Cytoscape. The constructed PPI network depicted in **Figure 9A** consisted of 142 interconnected nodes and 750 edges with an average local clustering coefficient of 0.452. The dense interconnectivity observed within the network suggests the potential therapeutic efficacy of NELNs on TNBC through coordinated modulation of multiple signaling pathways. Furthermore, to identify the key regulatory nodes involved in the PPI network, the top 15 hub targets were identified using CytoHubba in Cytoscape based on degree centrality (**Figure 9B**) indicating their pivotal roles in mediating compound–target interactions.

### 3.8 Functional enrichment and pathway analysis of potential hub targets

**Figure 10:**
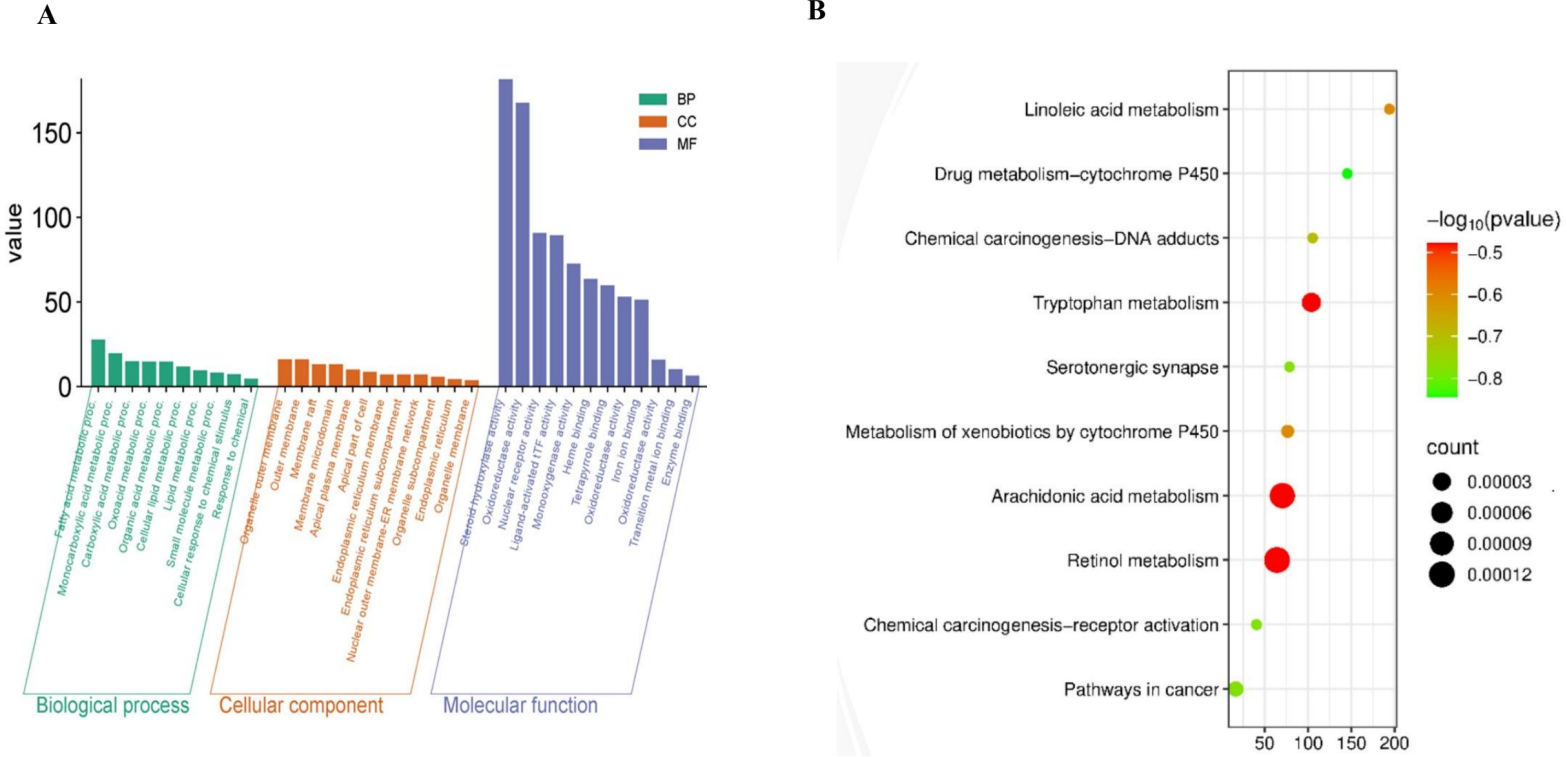
Gene ontology and KEGG pathway enrichment analysis of associated hub targets. **(A)** GO enrichment analysis categorized into three terms, namely, biological processes, cellular components, and molecular functions. **(B)** KEGG pathway enrichment analysis of NELN-associated hub targets.

To delineate the biological significance and functional enrichment of the identified hub targets, GO functional annotation and KEGG pathway enrichment analysis were performed. The enrichment analysis exhibited systematic insights into some key parameters such as BP, CC, MF, and pathway enrichment potentially regulated by NELN-encapsulated metabolites. As depicted in **Figure 10A**, the key biological processes enriched by GO functional annotation were predominantly associated with lipid metabolic processes, oxoacid metabolic processes, fatty acid metabolism, and cellular metabolic processes. In the CC category, target proteins were mostly localized to membrane-associated regions such as organelle outer membrane, apical plasma membrane, endoplasmic reticulum membrane, and intracellular membrane-bound compartments (**Figure 10A**). Additionally, enriched terms for molecular functions included oxidoreductase activity, monooxygenase activity, heme binding, tetrapyrrole binding, nuclear receptor activity, and ligand-activated transcription factor activity (**Figure 10A**). KEGG pathway enrichment analysis further revealed that among the enriched pathways, cytochrome P450-related pathways exhibited strong statistical significance and were recurrently represented across GO molecular function and KEGG analyses (**Figure 10B**). CYP1A2, CYP2C9, and CYP3A4 were selected for molecular docking as they were identified within the enriched cytochrome P450-associated pathways and are recognized as major enzymes involved in xenobiotic metabolism, carcinogen biotransformation, and metabolism of anticancer agents.

### 3.9 Molecular docking analysis and visualization of ligand–protein interaction

**Figure 11:**
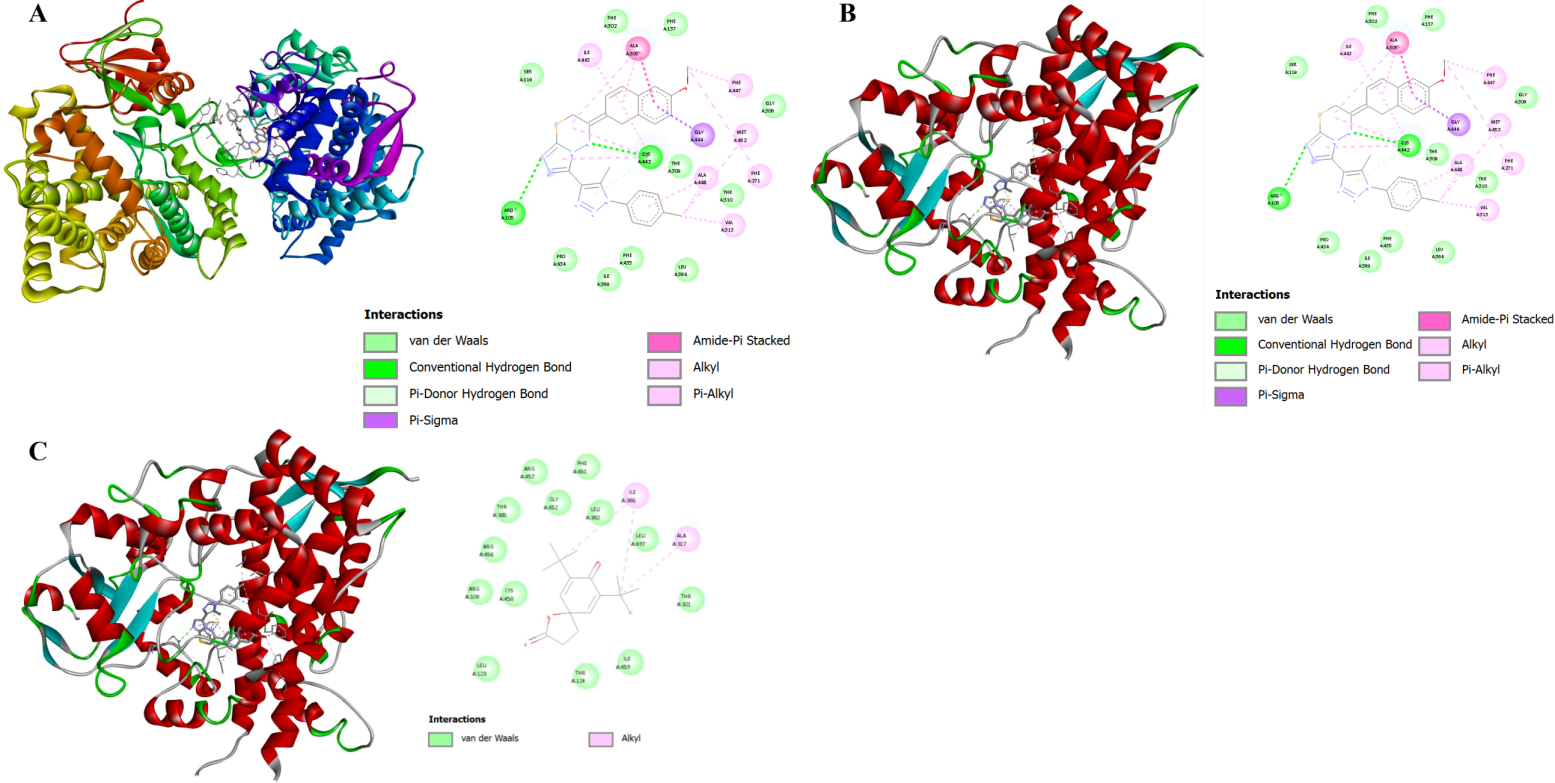
Visualization of 3D and 2D interactions of ligand–protein binding. **(A)** CYP2C9 docked with 7H-3-[5-methyl-1-(4-methylphenyl)-1,2,3-triazol-4-yl]-1,2,3-triazol-4-yl]-6-(6-methoxynaphthalen-2-yl)-s-triazolo[3,4-b]-1,3,4-thiadiazine. **(B)** CYP3A4 with 7H-3-[5-methyl-1-(4-methylphenyl)-1,2,3-triazol-4-yl]-1,2,3-triazol-4-yl]-6-(6-methoxynaphthalen-2-yl)-s-triazolo[3,4-b]-1,3,4-thiadiazine. **(C)** CYP1A2 with 7,9-di-tert-butyl-1-oxaspiro(4,5)deca-6,9-diene-2,8-dione.

Furthermore, to evaluate the interaction of NELN metabolites with the key cytochrome P450 proteins identified through network pharmacology studies, molecular docking analysis was performed for the NELN metabolites against CYP1A2, CYP2C9, and CYP3A4, and the binding affinities (kcal/mol) are reported in **Tables 1**–**3**, respectively. Among the different screened NELN metabolites, 2-acetyl-2-{bis[4-(dimethylamino)phenyl]methyl}cyclopentanone demonstrated the highest binding energy with the target proteins followed by 7,9-di-tert-butyl-1-oxaspiro(4,5)deca-6,9-diene-2,8-dione, phenol, 2,5-bis(1,1-dimethylethyl)-. Also, several long-chain hydrocarbons and fatty acid derivatives such as n-hexadecanoic acid, octadecane, and pentacosane also exhibited moderate binding affinity. Furthermore, the 2D and 3D images for the highest binding interactions were visualized using Discovery Studio and PyMol as depicted in **Figures 11A–C**.

**Table 1:** Binding energies (kcal/mol) of NELN-encapsulated metabolites with CYP1A2 (PDB ID: 2HI4).

Compounds	Binding energy (kcal/mol)
2-Acetyl-2-{bis[4-(dimethylamino)phenyl]methyl}cyclopentanone	−8.4
7,9-Di-tert-butyl-1-oxaspiro(4,5)deca-6,9-diene-2,8-dione	−8.0
Phenol, 2,5-bis(1,1-dimethylethyl)-	−7.2
Benzoic acid, 4-ethoxy-,ethyl ester	−6.8
Pentacosane	−6.4
n-Hexadecanoic acid	−6.3
Octadecane	−6.3
m-Xylene	−6.2
p-Xylene	−6.2
Hexadecanoic acid, methyl ester	−6.1
Cyclopropane, nonyl-	−6.0
Diethyl phthalate	−5.9
Tetradecanoic acid	−5.9
5-Nitro-2,4(1H,3H)-pyrimidinedione	−5.9
Undecane	−5.6
2-Propyl-tetrahydropyran-3-ol	−5.5
1,3,4,5-Tetrahydroxycyclohexanecarboxylic acid	−5.3
Phenol	−5.1
Toluene	−5.1
3-Amino-2-oxazolidinone	−4.2
Silanediol, dimethyl-	−3.1

**Table 2:** Binding energies (kcal/mol) of NELN-encapsulated metabolites with CYP2C9 (PDB ID: 4NZ2).

Compounds	Binding energy (kcal/mol)
2-Acetyl-2-{bis[4-(dimethylamino)phenyl]methyl}cyclopentanone	−7.4
7,9-Di-tert-butyl-1-oxaspiro(4,5)deca-6,9-diene-2,8-dione	−6.6
Phenol, 2,5-bis(1,1-dimethylethyl)-	−5.8
Diethyl phthalate	−5.8
Benzoic acid, 4-ethoxy-, ethyl ester	−5.8
n-Hexadecanoic acid	−5.6
Pentacosane	−5.5
p-Xylene	−5.4
Octadecane	−5.4
1,3,4,5-Tetrahydroxycyclohexanecarboxylic acid	−5.3
5-Nitro-2,4(1H,3H)-pyrimidinedione	−5.2
Tetradecanoic acid	−5.2
m-Xylene	−5.2
Hexadecanoic acid, methyl ester	−5.2
Cyclopropane, nonyl-	−4.8
2-Propyl-tetrahydropyran-3-ol	−4.8
Toluene	−4.7
Phenol	−4.7
Undecane	−4.5
3-Amino-2-oxazolidinone	−3.9
Silanediol, dimethyl-	−3.0

**Table 3:** Binding energies (kcal/mol) of NELN-encapsulated metabolites with CYP3A4 (PDB ID: 1TQN).

Compounds	Binding energy (kcal/mol)
2-Acetyl-2-{bis[4-(dimethylamino)phenyl]methyl}cyclopentanone	−7.6
7,9-Di-tert-butyl-1-oxaspiro(4,5)deca-6,9-diene-2,8-dione	−7.1
Diethyl phthalate	−6.7
Benzoic acid, 4-ethoxy-, ethyl ester	−6.6
Phenol, 2,5-bis(1,1-dimethylethyl)-	−6.5
Octadecane	−6.5
Pentacosane	−6.4
n-Hexadecanoic acid	−6.4
Tetradecanoic acid	−6.3
Cyclopropane, nonyl-	−6.2
Hexadecanoic acid, methyl ester	−6.0
Undecane	−5.9
m-Xylene	−5.9
p-Xylene	−5.9
1,3,4,5-Tetrahydroxycyclohexanecarboxylic acid	−5.6
5-Nitro-2,4(1H,3H)-pyrimidinedione	−5.5
2-Propyl-tetrahydropyran-3-ol	−5.3
Toluene	−5.3
Phenol	−4.9
3-Amino-2-oxazolidinone	−4.0
Silanediol, dimethyl-	−3.1

## 4 Discussion

The increasing emergence of drug resistance toward conventional therapies in cancer treatment highlights the urgent need for alternative and more effective therapeutic interventions in breast cancer. Although existing studies have demonstrated the anticancer efficacy of various plant extracts and their phytochemicals (55–57), their therapeutic efficacy is often limited given the complex phytochemical structure and their uptake into cancer cells. To address these limitations, there is a growing attention in the field of plant-derived exosome-like nanoparticles, which have exhibited promising anticancer potential and targeted delivery in breast cancer models (58–60). Plant-derived exosome-like nanoparticles have recently emerged as potential targeted cell-free therapeutics owing to their enhanced bioavailability and minimal toxicity. These extracellular vesicles are known to encapsulate diverse array of nucleic acids (such as DNA, mRNA, miRNA), proteins, lipids, and even phytochemicals enclosed by a phospholipid bilayer (61, 62) and upon cellular uptake can efficiently transfer their molecular cargoes into the recipient cells, thereby modulating the physiological process of the recipient cells. Interestingly, there are emerging studies reporting the selective uptake of plant ELNs into cancer cells, thus making them more efficient delivery systems in cancer therapy (63). Flowers possess an exquisite pool of enriched bioactive compounds potentially helpful in curing numerous ailments and diseases. Among the different flower species, *N. oleander* L. belonging to the family Apocynaceae has been widely studied in Ayurvedic remedies to have anti-inflammatory and anticancer potential (64). Consistent with the ethnopharmacological significance of *N. oleander* flowers, several studies have been documented on the efficacy of *N. oleander* leaves and flower extracts across different cancer models (65, 66). However, no studies till date have investigated the potential of NELNs as a therapeutic intervention in breast cancer studies. Therefore, the present study aimed to evaluate the potential biological activity of NELNs in TNBC treatment using MDA-MB-231 cell lines and to further delineate the molecular drivers of NELNs underlying their therapeutic activity through a network pharmacology approach.

For the isolation of NELNs from fresh *N. oleander* flowers, ultracentrifugation was used and the fresh NELN pellet was further dissolved in 1× PBS following previously documented protocols. Following the isolation of NELNs, they were further characterized using NTA and zeta potential analysis and visualized using FE-SEM. The concentration of isolated NELNs was approximately 5 × 10^13^ with particle sizes of 50–200 nm, which was consistent with the particle size and distribution of isolated ELNs in previously documented studies. Furthermore, visualization of NELNs through FE-SEM demonstrated a spherical vesicular morphology with a diameter of 180 nm. Additionally, it is important to analyze the stability of NELNs in solution, and to address that, we analyzed their zeta potential to evaluate the overall surface charge of the NELNs. We observed a zeta potential of −10 to −30 mV, demonstrating moderate to high stability of NELNs, summarizing that they would not form aggregates in solution. These findings are consistent with other previously reported results. However, it is important to note that while NTA and zeta potential provided information on particle size distribution, concentration, and colloidal stability, they fail to distinguish the purity of vesicles. Furthermore, FE-SEM confirmed the presence of nanoscale spherical structures; however, morphological assessment alone is insufficient to establish the biochemical composition or purity of the isolated particles. Future studies employing proteomic analyses, lipid profiling, and vesicle-associated marker characterization would further strengthen the identification and purity assessment of vesicles.

Selective cytotoxicity toward cancer cells represents a fundamental prerequisite in the development of anticancer therapeutics. To evaluate the cytotoxicity of NELNs in MDA-MB-231 cells, the MTT assay was performed and an effective inhibition of cancer cell proliferation was observed in a dose-dependent manner while exerting minimal cytotoxicity in the normal non-tumorigenic cell line HEK293T. This highlights the potential of NELNs in exerting cytotoxicity in cancerous cell lines, supporting their relevance in the development of cancer therapeutics. Furthermore, minimal cytotoxicity in HEK293T cells remains consistent with our recent findings on NELNs to exhibit lower cytotoxicity in RAW264.7 macrophages, supporting effective biocompatibility of NELNs (40). However, it is important to note that a broader assessment using other breast cancer cell lines and normal breast epithelial models is required to establish tumor selectivity. Additionally, our observation aligns with previous findings on demonstrating the anticancer potential of plant-derived ELNs. For instance, ELNs derived from garlic exhibited a reduction in cell viability across multiple cancer cell models including U-87 for glioblastoma, SH-SY5Y for neuroblastoma, and PC-3 for prostate cancer (67). Also, ELNs isolated from lemon have been shown to suppress the proliferation of gastric adenocarcinoma.

ROS generation in cancer cells is a critical mechanism underscoring the cytotoxic activity of several anticancer therapeutics as elevated oxidative stress leads to disruption of cellular redox homeostasis causing oxidative damage and resulting in cell death (68). Cancer cells usually maintain higher ROS levels in basal conditions due to higher metabolic reprogramming. Thus, the ability of NELNs to elevate intracellular ROS generation could lead to significant oxidative damage and cell death, rendering them potential therapeutics in TNBC treatment (69). Hence, in the present study, we have assessed the effect of NELNs on intracellular ROS generation of MDA-MB-231 cells using DCFDA staining through flow cytometry and fluorescence microscopy. Flow cytometric analysis revealed an increase in intracellular ROS levels post-NELN treatment compared to the untreated cells (**Figure 2C**). Furthermore, visualization through fluorescence microscopy demonstrated an increase in green fluorescence exhibited by NELN and doxorubicin treatment compared to the untreated cells depicted in **Figure 2D**, indicative of an elevated intracellular ROS generation. These findings are consistent with the emerging evidence of plant-derived ELNs in modulating intracellular signaling possibly through delivery of essential phytochemicals to the recipient cells capable of regulating oxidative stress.

Triggering apoptosis (programmed cell death) in cancer cells, along with suppressing other inflammatory damage related to necrosis, remains a fundamental strategy while developing various anticancer agents (70). In the present study, apoptosis was evaluated using AO/EtBr dual staining through fluorescence microscopy. Interestingly, it was observed that the NELN-treated cells exhibited yellowish and orangish fluorescence, indicative of alterations in membrane permeability, whereas untreated cells emitted green fluorescence, consistent with cell viability. Furthermore, we estimated the percentage of apoptotic cells using Annexin-PI through flow cytometry, and our results have demonstrated an increase in Annexin V-positive apoptotic cells post-NELN treatment compared to the untreated cells. The increase in percentage of early and late apoptotic cells is indicative of phosphatidyl serine externalization and loss of membrane integrity. Notably, both the positive control and NELN treatment did not induce any measurable necrotic cell death. However, it is important to acknowledge that the untreated control group exhibited a relatively elevated basal apoptotic fraction compared to typically observed healthy MDA-MB-231 cultures. This may partially reflect the intrinsic biology of this aggressive TNBC cell line, which is characterized by elevated basal oxidative stress, high metabolic activity, and genomic instability under *in vitro* culture conditions. Nevertheless, since all experimental groups were processed under identical conditions, the comparative increase in Annexin V-positive populations following NELN treatment remains indicative of enhanced apoptotic response. Also, induction of apoptosis remains consistent with elevated intracellular ROS levels following NELN treatment. Excessive ROS production can activate apoptotic pathways through disruption in mitochondrial membrane potential, cytochrome c release, and subsequent activation of caspases (69).

Cell cycle analysis is critical in analyzing cell proliferation and survival, and disruption of cell cycle represents a significant mechanism of anticancer therapeutics (71). In the present study, we have evaluated cell cycle distribution in MDA-MB-231 cells upon NELN treatment using PI staining through flow cytometric analysis. The untreated group exhibited a typical distribution of cells across various phases of the cell cycle with most of the cells accumulated in the G0–G1 phase followed by the S and M phases, while there was a smaller portion of cells present in the sub-G0 phase, representing basal apoptosis. On the contrary, NELN and positive control treatment resulted in an alteration of cell cycle progression. Notably, there was an increase in sub-G0 population, indicating enhanced DNA fragmentation and induction of apoptosis. Subsequently, there is a reduction of cell populations in the G0–G1, M, and S phases compared to the untreated group, suggesting an inhibition of DNA synthesis, thereby limiting cell proliferation. Collectively, both NELN and doxorubicin treatment exhibited an increased sub-G0 population with a decreased distribution across the other proliferative phases. Previous studies have suggested that doxorubicin is known to induce DNA-mediated cell cycle arrest and apoptosis through activation of checkpoint activation (72, 73). Interestingly, the similarity in cell cycle modulation between the observed effects of doxorubicin and NELNs suggests that NELNs exert cytotoxicity in MDA-MB-231 cells, possibly through DNA damage response pathways.

Furthermore, to elucidate the molecular mechanisms underlying the anticancer potential of NELNs in TNBC, we employed a network pharmacology approach integrating target prediction, PPI network, and functional enrichment analysis. The *in silico* target prediction using SEA and STP resulted in 174 overlapping targets relevant to NELNs, and furthermore, intersection of these 174 targets with TNBC-associated targets identified 142 common targets indicating that NELNs may exert regulatory effects through multiple targets. Furthermore, the PPI network was constructed and visualized using Cytoscape consisting of 142 nodes and 750 edges with a cluster coefficient of 0.452, indicating strong functional interconnectivity among the targets. Additionally, topological analysis revealed the top 15 hub genes primarily associated with inflammatory signaling, drug transport processes, xenobiotic metabolism, and nuclear receptor mechanism. Notably, among the hub genes, STAT3 and PTGS2 are well-known mediators for tumor progression in TNBC pathogenesis, suggesting that NELNs may potentially exert antiproliferative properties (74, 75). Furthermore, GO enrichment analysis further revealed the hub targets were predominantly associated with biological processes such as xenobiotic metabolism, oxidation–reduction mechanisms, and response to chemical stimuli, while cellular component analysis indicated localization within the endoplasmic reticulum membrane and microsomal compartments, consistent with the distribution of cytochrome P450 enzymes. Additionally, molecular function enrichment highlighted oxidoreductase activity, monooxygenase activity, heme binding, and drug binding, emphasizing the significance of metabolic and redox regulatory processes in the predicted mechanism of action for NELNs. Furthermore, KEGG pathway analysis revealed several enriched pathways, among which enrichment of cytochrome P450-related pathways suggests that NELNs may influence tumor-associated metabolic reprogramming and detoxification process. Among the identified hub targets, CYP1A2, CYP2C9, and CYP3A4 are important members of the cytochrome P450 superfamily that play critical roles in the metabolism of xenobiotics and various anticancer drugs (76, 77). Dysregulation of these enzymes has been mostly associated with altered carcinogen activation, oxidative stress, and metabolic reprogramming of cancer cells, thereby contributing to tumor initiation and progression (76). Moreover, previous reports have suggested that CYP protein-mediated metabolism can influence bioavailability and efficiency of chemotherapeutic drugs in TNBC, highlighting their importance in potential therapeutic targets (77, 78). To further analyze the interaction of NELN-encapsulated metabolites with these key CYP isoforms, molecular docking was performed against CYP1A2, CYP2C9, and CYP3A4. Notably, several metabolites exhibited favorable binding affinities with the target proteins, supporting their potential regulatory interactions. Notably, 2-acetyl-2-{bis[4-(dimethylamino)phenyl]methyl}cyclopentanone demonstrated the highest binding affinity against CYP1A2 (−8.4 kcal/mol), CYP2C9 (−7.4 kcal/mol), and CYP3A4 (−7.6 kcal/mol), indicating stable ligand–protein interaction across multiple CYP isoforms. Additionally, other metabolites have also demonstrated moderate to strong binding affinities as depicted in **Tables 1**–**3**.

The computational enrichment and molecular docking analyses suggest that metabolites previously identified from NELNs may potentially interact with CYP-associated pathways relevant to TNBC biology. However, these observations remain predictive and hypothesis-generating in nature, as intracellular metabolite uptake, CYP modulation, and downstream signaling effects were not experimentally validated in the present study. Therefore, further experimental investigations involving CYP expression/activity assays and pathway validation studies will be necessary to establish the biological relevance of the predicted interactions. Also, intracellular delivery of the identified metabolites into MDA-MB-231 cells under tested conditions and direct modulation of CYP signaling in TNBC have to be tested further. Also, it is important to note that although the network pharmacology analysis provided preliminary insights into potential bioactive compounds and molecular targets associated with the anticancer activity of NELNs, the metabolites used for the analysis were derived from our previous GC–MS study and were not directly characterized from the isolated NELNs used in the present investigation. Also, the identified compound–target interactions and associated pathways should be interpreted as predictive rather than definitive mechanistic evidence, which will require further *in vitro* and *in vivo* validation.

## 5 Conclusion

The present study demonstrates the anticancer potential of NELNs through a preliminary *in vitro* and *in silico* approach. Our findings demonstrated significant antiproliferative activity of NELNs in MDA-MB-231 cells under the tested condition, with comparatively minimal cytotoxicity observed in HEK293T cells. Moreover, NELNs exhibited elevated intracellular ROS generation, induced apoptosis, and modulated cell cycle progression in MDA-MB-231 cells. Furthermore, network pharmacology and molecular docking analyses generated putative mechanistic insights into pathways relevant to TNBC from the perspective of NELN-encapsulated metabolites. However, these computational predictions require further experimental validation to establish the biological relevance of CYP-associated interactions and signaling mechanisms. Nevertheless, further *in vivo* validation in TNBC animal models and detailed mechanistic studies are essential to elucidate the role of cytochrome P450 modulation along with establishing the translational relevance of NELNs in TNBC treatment.

## Data Availability

The original contributions presented in the study are included in the article/supplementary material. Further inquiries can be directed to the corresponding author.
